# Translational switching from growth to defense – a common role for TOR in plant and mammalian immunity?

**DOI:** 10.1093/jxb/erx120

**Published:** 2017-05-30

**Authors:** María Eugenia Zanetti, Flavio A. Blanco

**Affiliations:** 1 Instituto de Biotecnología y Biología Molecular, Facultad de Ciencias Exactas, Universidad Nacional de La Plata, CCT-La Plata, CONICET, La Plata, Argentina

**Keywords:** Effector-triggered immunity (ETI), NB-LRR proteins, plant defense, target of rapamycin (TOR), translation.

## Abstract

This article comments on:

Meteignier LV, El Oirdi M, Cohen M, Barff T, Matteau D, Lucier JF, Rodrigue S, Jacques PE, Yoshioka K, Moffett P. 2017. Translatome analysis of an NB-1 LRR immune response identifies important contributors to plant immunity in Arabidopsis. Journal of Experimental Botany 68, 2077–2081.

**Characterization of mRNA populations associated with the translational machinery (translatome) is shedding light on the molecular mechanisms of plant environmental responses. The work presented by Meteignier *et al.* (2017) describes how selective changes in translation modulate the transition from growth to defense responses in Arabidopsis, revealing new similarities between plant and animal immunity.**

Plants have developed sophisticated mechanisms to adjust their developmental programs in response to changes in their environment. This adaptation largely depends on selective changes in gene expression, which include transcriptional and post-transcriptional control. Transcriptional changes provide a long-term response and, usually, are triggered by a signaling pathway initiated by signal perception that culminates in the activation of transcription factors in the nucleus. Transcriptome (the population of total cellular mRNAs) studies have enabled the identification of genes that are crucial for adaptation in numerous plant species. However, mRNA abundance and protein levels do not always correlate due to co- and post-transcriptional mechanisms controlling gene expression. Among such post-transcriptional mechanisms, mRNA translation plays a crucial role in controlling the amount of protein present in a cell or tissue. Translational control has been observed in a number of developmental processes in plants, as well as in response to environmental cues.

During the past decade, an increasing number of studies have focused on changes in the translatome (the population of actively translating mRNAs) during phase-transitions or perturbation caused by endogenous or exogenous signals. From these studies we know that translational control can be global, affecting most cellular mRNAs, or selective, affecting just a subset. Global translational repression has been observed during stresses that produce a cellular ‘energy crisis’, such as hypoxia (Branco-Price *et al.*, 2005; Branco-Price *et al.*, 2008; Mustroph *et al.*, 2009), heat (Yanguez *et al.*, 2013), and drought (Kawaguchi and Bailey-Serres, 2002; Kawaguchi *et al.*, 2004; Lei *et al.*, 2015). On the other hand, selective translational regulation has been associated with dark/light transitions (Juntawong and Bailey-Serres, 2012), photomorphogenesis (Liu *et al.*, 2013), daily clock cycles (Missra *et al.*, 2015), and symbiosis with nitrogen-fixing bacteria (Reynoso *et al.*, 2013).

Meteignier *et al.* (2017) show that selective translational control also occurs during plant immunity. A remarkable characteristic of translational regulation is that it enables rapid adjustment of the proteome using the existing transcriptome, thus providing cells or tissues with a fast and flexible response for adapting to changes in their environment, as in the case of the hypersensitive response triggered by some plant pathogens. This rapid response is frequently achieved by controlling the initiation step of translation, i.e. by increasing or decreasing the number of molecules of individual transcripts that are recruited to the translational machinery without a change in transcript abundance or even, in some cases, with an opposite change in transcript abundance.

## Translational control of immunity in plants and animals

Plant immunity against pathogens relies on mechanisms that allow host cells to perceive pathogens and mount a rapid and effective response that limits their spread. The work of Meteignier *et al.* (2017) used TRAP-seq technology (Box 1) to characterize the rapid and selective translational response of Arabidopsis plants upon activation of the effector-triggered immunity (ETI) response. The authors found that, during short intervals following ETI activation, the translational and transcriptional responses were uncoupled, especially for many of the genes that are negatively regulated. Accordingly, those genes with uncoupled transcription/translation showed the larger changes in translational efficiency upon activation of ETI. Genes involved in photosynthesis, gravi- and photo-tropism, auxin metabolism and carbohydrate biosynthesis were down-regulated at the translational level, whereas genes that participate in cell wall thickness, secondary metabolism and cell death were translationally up-regulated, revealing a reprogramming of the host cells, which appear to undergo a switch from growth and development to defense (Box 2).

**Box 1. TRAP-seq methodology**

Pioneer translatome studies used ultracentrifugation through sucrose-density gradients to fractionate 40S and 60S ribosomal subunits, 80S monosomes and polyribosomes (polysomes) of different size (mRNAs with ≥2 ribosomes). Later on, a method based on affinity purification known as TRAP (Translating Ribosome Affinity Purification) was developed for plants (Zanetti *et al.*, 2005), mammals (Heiman *et al.*, 2008) and yeast (Halbeisen and Gerber, 2009). In plants, TRAP-seq is based on the expression of FLAG-tagged Ribosomal Protein Large subunit 18 (RPL18), which is incorporated into translating ribosomes, providing a means of affinity-purifying ribosomes, polysomes and associated mRNAs. The population of mRNAs present in TRAP samples is then analyzed by direct RNA sequencing. In parallel, total cellular mRNAs from the same tissue and conditions are analyzed by RNA-seq, thus allowing a comparison of transcriptional and translational changes in gene expression, as well as an estimation of the translational efficiency (the Log_2_ ratio of the amount of polysome-associated RNA to the total cellular amount of RNA for a given mRNA). More recently, a technique that precisely maps the ribosome-protected regions in an individual mRNA, known as ribosome footprinting or ribosome profiling (Ingolia *et al.*, 2009), has been combined with TRAP and applied to gain insights into mechanistic aspects of translational control in plants (Juntawong *et al.*, 2014).

**Figure:**
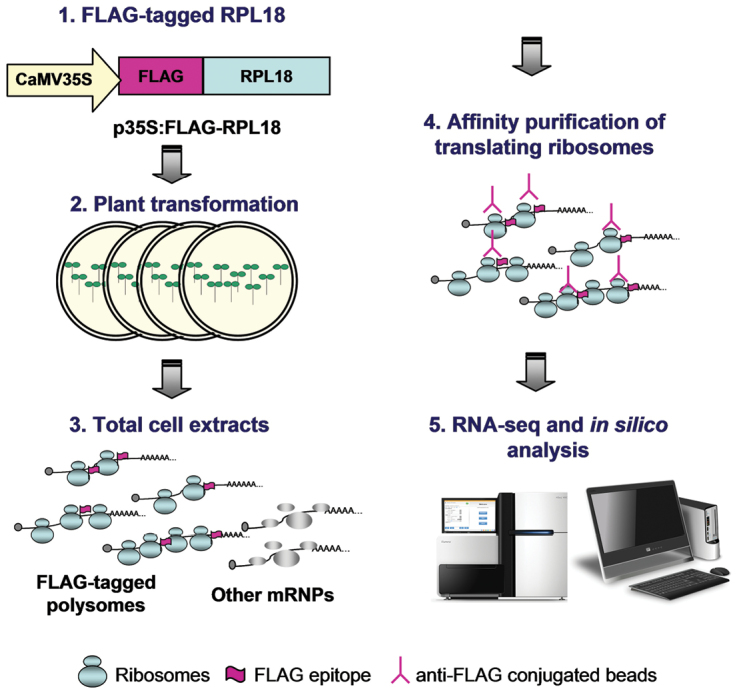

**Box 2. Transcriptional and translational changes mediated by activation of NB-LRR receptors during the ETI response**

Plant defense is based on two recognition systems. Pattern recognition receptors (PRRs) recognize pathogen-associated molecular patterns (PAMPs, such as the flagellin protein) to induce PAMP-triggered immunity (PTI), a level of defense that can be overcome by the production of pathogen effectors, such as avrRpm1, which is produced by *Pseudomonas syringae* and introduced into the plant cell by the type III secretion system. The second layer of defense relies on the recognition of these effectors by receptors, such as the NB-LRR RPM1 of Arabidopsis, triggering effector-triggered immunity (ETI), which induces a similar response to PTI, but more quickly and robustly. ETI can lead to the hypersensitive response (HR), a form of cell death limiting the spread of bio- and hemibio-trophic pathogens. Signaling events down-stream of receptor activation are poorly understood, but they include modulation of transcriptional responses in the nucleus. Meteignier *et al.* (2017) reveal that ETI also leads to changes in the translational status of pre-existing mRNAs in the cytoplasm. Translationally regulated mRNAs include those encoding HR-associated proteins, Target of Rapamycin (TOR) and other proteins such as Phosphorylcholine cytidylyltransferase 2 (CCT2), the calossin-like protein BIG and CBL-Interacting protein kinase 5 (CIPK5). TOR seems to affect the transition from growth to defense, whereas the other genes might act as positive or negative regulators of defense responses.

**Figure:**
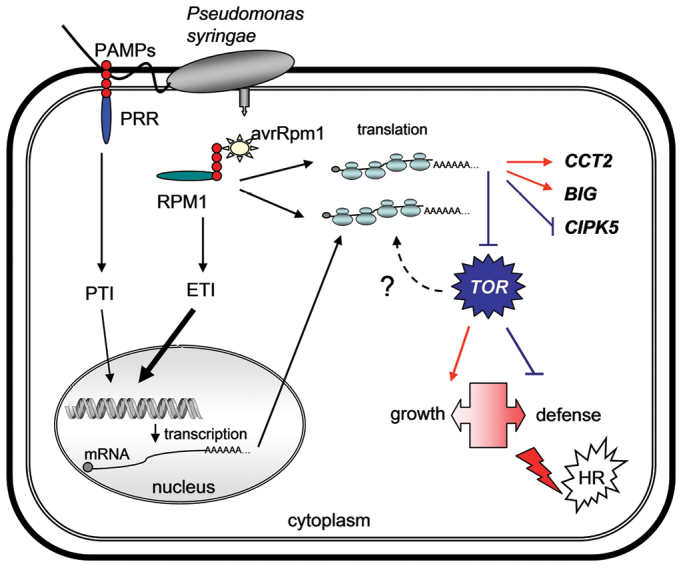

A number of novel genes involved in plant immunity were identified through this translatomic analysis, including Phosphorylcholine cytidylyltransferase 2 (CCT2), the calossin-like protein BIG, and CBL-Interacting protein kinase 5 (CIPK5), and these were functionally validated by reverse genetics. Interestingly, Target of Rapamycin (TOR), which regulates plant development by restructuring cell growth and carbon/nitrogen metabolism (Ren *et al.*, 2012), dramatically decreased its translational efficiency upon activation of ETI. Moreover, Meteignier and co-workers showed that silencing TOR decreased plant susceptibility to the pathogenic bacterium *Pseudomonas syringae* and the oomycete *Hyaloperonospora arabidopsidis*, suggesting that TOR acts as a negative regulator of disease resistance. Previous studies have shown that the transcriptome of plants with reduced TOR activity is enriched in transcripts involved in defense responses and innate immunity, supporting the notion that TOR negatively regulates defense responses. This suggest that low levels of TOR are associated with reprogramming the growth-to-defense transition. In addition, reduction of TOR activity decreased anabolism and increased catabolism (Caldana *et al.*, 2013), which might be linked to the reorientation of cellular activities to generate the energy required to mount a defense response.

The identification of TOR as a negative regulator of plant immunity has novel implications concerning the similarities between plant and animal immune systems. In mammals, the TOR pathway integrates exogenous signals with endogenous cues such as cellular energy status, hormones and growth factors to stimulate cell growth, proliferation and differentiation. This pathway also plays a central role in the translational control of innate or adaptive immunity (Piccirillo *et al.*, 2014). The best-characterized translational targets of TOR in mammals are 4E-binding proteins (4E-BPs), which act as translational suppressors by binding the eukaryotic initiation factor 4E (eIF4E, the cap-binding subunit of the eIF4F complex), preventing the formation of the eIF4F complex. The TOR–4EBPeIF4E module influences the translation of a subset of mRNAs referred to as ‘eIF4E sensitive’, including those encoding proteins involved in immunity, such as the transcription factor interferon regulatory factor 7 (IRF7). Mice deficient in 4EBPs are less susceptible than the wild type to viral infection and unable to suppress the translation of the IRF7 transcript upon virus-induced inhibition of TOR signaling (Colina *et al.*, 2008). Although the translational targets of TOR signaling in plants are not known yet, this pathway seems to play an active role in the switch from growth to immunity in both plants and mammals. Further studies are required to elucidate whether TOR influences the selective translation of mRNAs during plant immunity and the potential targets of translational control of this pathway in plants.

## Looking forward: mechanistic insights into translational control in plants

Most of the high-throughput analyses have centered on the characterization of transcriptomes, but application of new techniques, such as TRAP-seq and Ribo-seq, have started to reveal the importance of translation in the regulation of gene expression. The study of Meteignier *et al.* nicely illustrates how the analysis of translatomes might contribute to the identification of new players with important functions in the adaptation and response of plants to environmental challenges. Many biological processes could be fully understood only through the characterization of gene expression changes at different levels, complementing transcriptome studies with the analysis of translatomes, ribosome footprints, degradomes and proteomes. It is expected that the partial contribution of each regulatory level will be different for each process depending on the complexity and the time of the response. Rapid responses, such as the hypersensitive response, seem to rely on changes at the post-transcriptional level, activating the translation of pre-existing mRNAs that are stored inside the cell such that they are ready at the moment of challenge, generating a functional protein in a short time-frame. In addition, it is necessary to study these processes in the individual cell types involved in the response, which can be achieved by combining TRAP with the use of cell-specific promoters (Mustroph *et al.*, 2009). Also, to understand better how genes are regulated at the translational level it will be crucial to identify the *cis*-regulatory elements present in each transcript, as well as the *trans*-acting RNA-binding protein, which influence its selective association with the translational machinery and, more importantly, how its translational status can be modulated to generate the dynamic response required to adapt rapidly to changes in the environment. A preliminary study by Meteignier, Moffett and colleagues analyzing their candidates for the presence of shared elements in the untranslated regions is now available (Munusamy *et al.*, 2017).
